# Deep eutectic solvent extraction and biological activity of polysaccharides from *Tenebrio molitor*

**DOI:** 10.1016/j.heliyon.2025.e41790

**Published:** 2025-01-11

**Authors:** Shengru Yang, Xu Li, Qiaoli Li

**Affiliations:** The First Affiliated Hospital of Henan University, Kaifeng, 475000, PR China

**Keywords:** Polysaccharides, *Tenebrio molitor*, Antioxidant, Response surface, Deep eutectic solvents

## Abstract

This study aimed to extract polysaccharides from *Tenebrio molitor* using ultrasound-assisted deep eutectic solvent (DESs) and to evaluate their structural features, as well as their antimicrobial, antioxidant, and α-amylase inhibitory activities. Various DESs were tested for polysaccharides extraction, and the process was optimized using response surface methodology (RSM). A preliminary structural analysis of the polysaccharides was conducted using infrared spectrum. The DESs were characterized by measuring their pH, viscosity, conductivity, refractive index, and density. The optimal extraction agent and parameters were determined. Significant differences in pH, viscosity, and conductivity were observed among DESs, whereas differences in refractive index and density were not significant. Choline chloride-lactic acid was identified as the optimal extraction agent. The optimal extraction parameters were a DES molar ratio of 1:2.1, a water content of 27 %, an extraction temperature of 70 °C, and an extraction time of 44 min, resulting in polysaccharides yield of 18.62 %. The extracted polysaccharides exhibited strong inhibitory effects against Salmonella, along with antioxidant activity and α-amylase inhibitory activities. The study demonstrated that polysaccharides from *Tenebrio molitor* can be efficiently extracted using DESs, showcasing significant biological activities, including antibacterial, antioxidant, and α-amylase inhibitory properties. These findings highlight the potential applications of *Tenebrio****molitor*** polysaccharides as valuable biological resources.

## 1 Introduction

*Tenebrio molitor* is hailed as a "treasure trove of protein feed" due to its high nutritional content, which includes various bioactive substances such as antioxidative peptides, antifreeze proteins, antimicrobial peptides, and chitosan, all capable of eliminating free radicals in organisms [1]. With its low breeding costs and high fat content, which provides essential fatty acids beneficial to human health, *T. molitor* represents an insect resource with immense development potential [2].

Polysaccharides are widely distributed natural high-molecular-weight compounds that are closely associated with mechanisms essential for maintaining biological functions. They exhibit a wide range of biological activities [3] and have extensive applications in the food and pharmaceutical industries. Most polysaccharides demonstrate potent pharmacological effects, including anti-tumor, antioxidative, anti-inflammatory, and immunomodulatory properties, making them valuable sources for developing functional foods and medicines [4]. As an active and efficient component of *T. molitor*, polysaccharides hold significant potential for further development and application. Additionally, Studies have shown that incorporating natural preservatives into food can effectively extend its shelf life [5]. Chitosan, renowned for its diverse biological activities, broad availability, biocompatibility, and non-toxic nature, has emerged as a prominent topic in food research [6]. Currently, the extraction methods for polysaccharides in the food industry primarily include traditional solvent extraction and high-temperature water extraction. In comparison, Ultrasound-assisted extraction leverages the mechanical and cavitation effects of ultrasound to disrupt biological structures such as cell walls and cell membranes. The disruption of these structures facilitates solvent penetration into the material, thereby improving the extraction efficiency of bioactive compounds [7], as a result, ultrasound-assisted extraction offers several advantages, including shorter extraction times, operational simplicity, and higher extraction yields [[8], [9], [10]]. whereas high-temperature water extraction is time-consuming, requires large amounts of solvents, and results in lower extraction yields [11], it no longer meets the demands of the market.

Response surface methodology (RSM) is a comprehensive approach that combines experimental design and mathematical modeling for process optimization. It establishes functional relationships between independent factors and response values through well-structured experimental designs and appropriate data processing, ultimately yielding a regression equation. By analyzing this regression equation, the optimal process parameters can be identified [12]. One of the primary advantages of RSM is its ability to accurately explore the relationships between factors and response values while minimizing the need for extensive experimentation [13,14].

DESs are considered green solvents due to their minimal environmental impact, easy availability, low toxicity, and cost-effectiveness. Compared to other organic solvents, they exhibit superior solubility and diffusion properties [[15], [16], [17]]. Consequently, the use of ultrasound-assisted DESs for polysaccharides extraction aligns with the future demands of the food industry. During the extraction process, the viscosity, diffusivity, and solubility of DESs vary, influencing their ability to dissolve various polysaccharides [18,19]. This underscores the necessity of further exploration into these solvent systems.

DESs are innovative extraction solvents [20], composed of hydrogen bond acceptors (HBAs) and hydrogen bond donors (HBDs). The formation of hydrogen bonds between these components results in a low melting point and excellent solubilization properties, enabling DESs to efficiently extract active compounds from plants. For instance, hydrogen bond donors, such as alcohols, carboxylic acids, or polyalcohols, can form hydrogen bonds with phenolic compounds, including flavonoids and phenolic acids, thereby significantly facilitating their solubilization and extraction [21,22]. Various DES systems, such as combinations of choline chloride with different HBDs, have been utilized to extract a wide range of plant constituents, such as flavonoids, coumarins, and polysaccharides. These systems often outperform conventional solvents, achieving superior extraction efficiency and selectivity [23]. In recent years, DESs have demonstrated unique benefits in the extraction and separation of natural active ingredients [24,25]. As emerging solvents, they have been widely applied in the pharmaceutical, chemical, and food industries for separation and synthesis [26,27]. To enhance the extraction efficiency of *T. molitor* polysaccharides and improve resource utilization, this study focuses on the ultrasound-assisted extraction of *T. molitor* polysaccharides using DESs and investigates their biological activities. The goal is to provide a reference for the further development and utilization of *T. molitor* polysaccharides products.

## 2 Materials and methods

### 2.1 Materials

*T. molitor* was purchased from Aijia Pet Aquarium Supplies Co., Ltd. (Shanghai, China). 2,2′-Biazobis-3-ethylbenzothiazoline-6-sulfonic acid was purchased from Shanghai McLean Biotech Co., Ltd. (Shanghai, China). 1,1-Diphenyl-2-picrylhydrazyl was purchased from Shandong Xiya Chemical Co., Ltd. (Shandong, China). Ascorbic acid was purchased from Tianjin Damao Chemical Reagent Factory (Tianjin, China). All other reagents used were of analytical grade.

### 2.2 Screening and physical properties of DESs

#### 2.2.1 Sample preparation

The dried *T. molitor* was crushed and degreased using petroleum ether with a material-to-liquid ratio of 1:8, a degreasing time of 5 h, and a temperature of 75 °C under water bath heating. After degreasing, the mixture was allowed to stand for stratification. The supernatant was discarded, and the remaining *T. molitor* powder was dried in an oven. The dried powder was sieved through a 100-mesh sieve and stored in a sealed bag at a low temperature.

#### 2.2.2 DESs preparation

DESs were prepared using the heating method [28]. According to the parameters listed in Table 1, the components were mixed and placed in a 500 mL conical flask. The mixture was stirred with a magnetic stirrer at 80 °C for 30 min. After stirring, the mixture became clear and transparent and was set aside for further use.

**Table 1:** Components of different DES types.

serial number	hydrogen bond receptor	hydrogen bond receptor	molar ratio	moisture content
DES-1	Choline chloride	urea (NH2)2CO	1:2	30 %
DES-2	Choline chloride	Propanetriol	1:2	30 %
DES-3	Choline chloride	Isopropanol	1:2	30 %
DES-4	Choline chloride	Lactic acid	1:2	30 %
DES-5	Choline chloride	Citric acid	1:2	30 %
DES-6	Choline chloride	Trifluoroacetic acid	1:2	30 %

#### 2.2.3 Screening of the DESs

##### 2.2.3.1 Extraction of polysaccharides from *T. molitor* using the DES method

1.0 g of defatted *T. molitor* powder was processed with six distinct types of Deep Eutectic Solvents (DES) at a molar ratio of 1:2, a water content of 30 %, and a material-liquid ratio of 1:30. The mixture was subjected to ultrasonication at 80 °C for 40 min, followed by centrifugation at 5000 rpm for 15 min. The resulting supernatant was filtered using a vacuum pump and set aside for further use.

##### 2.2.3.2 Determination of polysaccharides by the phenol sulfuric acid method

The determination of polysaccharides followed the method described by Tatsuya Masuko et al. [29]. Briefly, the sample reacts with phenol and concentrated sulfuric acid, resulting in a color change. The absorbance was measured at 490 nm to calculate the polysaccharides concentration. The polysaccharide yield was calculated as Y (%) = c/w × 100 %, where c is the mass of polysaccharide and w is the mass of dried extract.

#### 2.2.4 Extraction methods and process parameter optimization

##### 2.2.4.1 Extraction of polysaccharides from *T. molitor* using hot water

1.0 g of *T. molitor* powder and 20 mL of distilled water were placed in a 250 mL conical flask. The ultrasonication was performed at 80 °C for 40 min. After ultrasonication, the mixture was centrifuged at 4000 rpm for 15 min. The supernatant was collected to calculate the polysaccharides yield.

##### 2.2.4.2 Extraction of polysaccharides from *T. molitor* using the DES hot water

1.0 g of *T. molitor* powder was mixed with 30 mL of choline chloride-lactic acid (molar ratio 1:2, 30 % water content) in a 250 mL conical flask. The reaction was carried out in a water bath at 50 °C for 40 min. After the reaction, the mixture was centrifuged at 4000 rpm for 15 min. The supernatant was collected, vacuum filtration, and used for polysaccharides yield measurement.

##### 2.2.4.3 Ultrasound-assisted extraction of polysaccharides from *T. molitor* using DES

Based on DES thermal leaching, ultrasound-assisted DES was used to extract polysaccharides from *T. molitor*, with ultrasonic temperatures set at 50 °C, 60 °C, 70 °C, and 80 °C, respectively. After ultrasonication, the mixture was centrifuged at 4000 rpm for 15 min. The supernatant was then collected for vacuum filtration, and the polysaccharides yield at different temperatures was calculated. The optimal extraction temperature parameters for the single-factor experiment were determined from these results.

#### 2.2.5 Physical properties of DESs

##### 2.2.5.1 pH

The pH of the DES samples was measured using a pH meter. The meter was first calibrated with two standard buffers: potassium hydrogen phthalate (pH = 4.00) and potassium dihydrogen phosphate (pH = 6.86). An appropriate volume of each of the six DES (prepared with a molar ratio of 1:2 and 30 % water content) was placed in a beaker, and the pH was recorded.

##### 2.2.5.2 Viscosity

The viscosity of the six DES samples was measured using a viscometer. An appropriate volume of each DES was placed in a beaker, and a rotor with a speed setting of 12 rpm was selected. The percentage reading was maintained within the range of 10 %–90 %, and the rotor was allowed to remain in the solution for 2–3 min before recording the value.

##### 2.2.5.3 Conductivity

The conductivity of the samples was measured using a conductivity meter. An appropriate volume of each of the six DES was first placed in a beaker. The Burr Black 10 electrode was used, and the meter was calibrated with a 0.1 mol/L potassium chloride standard solution. The conductivity of each sample was then measured and recorded.

##### 2.2.5.4 Refractive

The refractive index of the test samples was measured using an Abbe refractometer. The refractometer was first calibrated with distilled water. The knob was adjusted until the critical line in the field of view aligned precisely with the intersection of the X-shaped crosshairs. After calibration, a suitable volume of each of the six types of DES was prepared. Any residual distilled water on the prism was wiped off, and two to three drops of the test sample were uniformly spread onto the prism. The knob was then adjusted to align the critical line with the intersection, and the corresponding values were recorded.

##### 2.2.5.5 Density

The density was measured using a temperature-controlled pycnometer. The pycnometer was first washed with distilled water, rinsed with ether and ethanol, and thoroughly dried. The empty pycnometer was weighed, and its weight was recorded as G_1_. Distilled water was then added to fill the pycnometer, ensuring no air bubbles were present. Excess water was carefully removed using filter paper, and the pycnometer was sealed. The sealed pycnometer was weighed, and its weight was recorded as G_3._ After emptying the distilled water, the pycnometer was dried again, and the sample to be measured was added. The same procedure was followed, and the combined weight of the pycnometer and the sample was recorded as G2. The density of the sample was then calculated using Equation (1).

(1) $$\text{Sample}\;\text{density}=\frac{G_{2}‐G_{1}}{G_{3}‐G_{1}}$$

### 2.3 Single-factor experiment for polysaccharides extraction from the *T. molitor*

Five portions of defatted *T. molitor* powder were weighed and added to choline chloride-lactic acid mixtures with varying conditions: DES water content (10 %, 20 %, 30 %, 40 %, 50 %), molar ratio (2:1, 1:1, 1:2, 1:3, 1:4), and material-liquid ratio (1:20, 1:25, 1:30, 1:35, 1:40). The ultrasonic temperature was set at 60 °C, 70 °C, 80 °C, 90 °C, 100 °C, with a power of 200W. Under these conditions, the ultrasonic time was adjusted to 20, 30, 40, 50, and 60 min. After ultrasonication, the supernatant was collected, vacuum filtered, and diluted to 100 times its original volume. Subsequently, 2 mL of the diluted sample solution was mixed with 1 mL of 5 % phenol and 5 mL of concentrated sulfuric acid. The mixture was homogenized and left to stand for 20 min. The absorbance was then measured at 490 nm. Using the polysaccharides content as the response value, the absorbance values of the extracts under different conditions were determined, and the polysaccharides yield was calculated.

### 2.4 The experiment of response surface

After analyzing the significance and variance of the results from the single-factor experiment, three factors were selected: sonication time, DES molar ratio, and DES water content. The response variable was the polysaccharides yield. These factors were optimized using response surface methodology at three levels. The 17 sets of data generated by the software were tested individually, and the results were analyzed. Ultimately, the optimal process conditions for ultrasound-assisted DES extraction of polysaccharides from *T. molitor* were determined [30]. The selected factors and levels selected were shown in Table 2.

**Table 2:** The analysis factors and levels of response surface.

level	An Ultrasonic Time/min	B Molar Ratio	C Water content/%
1	30	1:1	20 %
0	40	1:2	30 %
−1	50	1:3	40 %

### 2.5 Separation and purification of *T. molitor* polysaccharides

**Fig. 1:**
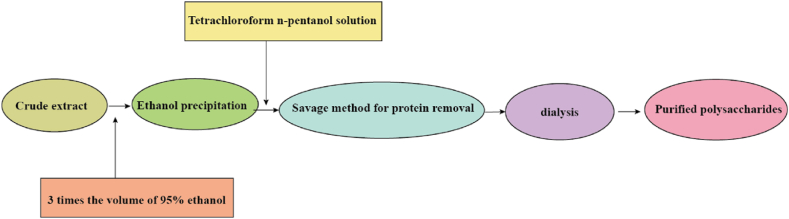
Extraction liquid separation and purification process diagram.

The *T. molitor* polysaccharides extracted using the DESs required preliminary separation and purification, after which the biological activity of the purified polysaccharides was studied. The processes for separation and purification were shown in Fig. 1.

### 2.6 Infrared spectroscopy analysis of *T. molitor* polysaccharides

Preliminarily purified samples were dried in an oven at 45–50 °C for 2 h. An appropriate amount of potassium bromide was also dried in an oven at 105 °C for 2–3 h. 3 mg of the purified polysaccharides powder and 100 mg of potassium bromide were weighed, mixed, and ground into a fine powder in an agate mortar under a far-infrared lamp. The mixture was then pressed into tablets and scanned using an infrared spectrometer with a wavelength range of 4000–500 cm^−1^, and the spectral peaks were recorded. Additionally, 100 mg of potassium bromide were ground and pressed into tablets. An appropriate amount of choline chloride-lactic acid was diluted with anhydrous ethanol, and 1–2 drops of the diluted solvent were added to the pressed tablets. these were then baked under far-infrared lamp, and the procedure was repeated for control. The spectral peaks were observed for comparison.

### 2.7 Antibacterial activity of *T. molitor* polysaccharides

#### 2.7.1 Experimental pre-processing

##### 2.7.1.1 Isolation and purification of strains

several 9 mL tubes of sterile water were prepared, and all procedures were performed in a laminar flow cabinet. Strains were Transferred from frozen tubes into the sterile water using an inoculation loop, mixed thoroughly, and 1 mL of this solution was pipetted into another sterile water tube. This process was repeat five times to obtain bacterial suspensions with concentration gradients of 10^−1^, 10^−2^, 10^−3^, 10^−4^, and 10^−5^. Then 0.2 mL of each suspension was pipetted, spread evenly on agar plates, and the plates were labeled with a marker. Fungi were incubated at 28 °C for 48 h, and bacteria were incubated at 37 °C for 24 h. After incubation, plates with isolated colonies were selected, microscopic examinations were performed to confirm pure strains, and these strains were transferred to slant media for low-temperature preservation.

##### 2.7.1.2 Preparation of bacterial solutions

In a sterile environment, the strains were inoculated into a liquid medium. The bacterial cultures were incubated in a shaker at 37 °C for 24 h, while the fungal cultures were incubated in a shaker at 28 °C for 48 h. The cultures were then set aside for further use.

#### 2.7.2 Minimum inhibitory concentration (MIC) and antimicrobial properties of *T. molitor* polysaccharides

##### 2.7.2.1 Pre-treatment of experimental materials

First, filter paper sheets, microfiltration membranes, petri dishes, and other items were wrapped in newspaper and sterilized in an autoclave. Next, the required supplies were placed on the ultra-clean bench, ensuring the UV lamps in aseptic room had been turned on for 1h before the start of the experiment. This step was intended to minimize the presence of hybrid bacteria that could contaminate the environment during the experiment. Finally, the solid medium was heated until it reached a liquid state and set aside.

##### 2.7.2.2 The pre-treatment of samples

Polysaccharides solutions with concentrations of 10, 8, 6, 4, 2, 1, and 0.5 mg/mL were prepared and set aside. Each sample solution was Filtered through a 0.22 μm microfiltration membrane to remove bacteria. The filtered sample solution was then dripped into pre-prepared test tubes containing an appropriate amount of filter paper until the sheets were completely submerged. Each concentration of the polysaccharides solution was processed sequentially as described, the test tubes were labeled, and the filter paper sheets were immersed for 30–40 min.

##### 2.7.2.3 Determination of MIC

A 0.2 mL aliquot of bacterial suspension was evenly spread onto solidified agar plates. Filter paper discs, pre-soaked in the test solutions and allowed to dry to a non-dripping state, were carefully placed onto the agar plates using tweezers (two discs per plate). Sterile water was used as the negative control, while a 6 mg/mL solution of penicillin sodium served as the positive control. After incubation, the diameters of the inhibition zones were measured using the crossover method. The experiment was conducted in triplicate, and the average values were calculated. The concentration at which no inhibition zone was observed was defined as the MIC of *T. molitor* polysaccharides against the strain. The antimicrobial properties of *T. molitor* polysaccharides were assessed based on the size of the inhibition zones.

### 2.8 Antioxidant activity of *T. molitor* polysaccharides

An appropriate amount of purified and dried polysaccharides powder was dissolved in water and then diluted with distilled water to concentrations of 0.2, 0.4, 0.6, 0.8, 1.0, and 1.2 mg/mL. These solutions were set aside for use. Ascorbic acid (V_C_) solutions with the same concentration gradient as the polysaccharides solution was prepared to serve as positive controls. The scavenging rates of the samples and V_C_ were calculated according to Equation (2).

(2) $$\text{Radical}\;\text{scavenging}\left(\%\right)=\frac{A_{0}‐A_{1}+A_{2}}{A_{0}}\times 100$$

Where: A_0_ Absorbance of distilled water or anhydrous ethanol (blank control group); A_1_ Absorbance of yellow powdery mildew polysaccharide solution (reaction group); A_2_ Absorbance of vitamin C solution (control group).

#### 2.8.1 DPPH radical scavenging activity

The DPPH radical scavenging activity was measured using the methods previously described by Ren et al. [31]. 2.0 mL of polysaccharides solution was added to a test tube, followed by 3.0 mL of 0.5 mmol/L DPPH-ethanol solution. The mixture was shaken and incubated in the dark for 30 min. After the reaction, the absorbance was measured at 517 nm and recorded [32]. Anhydrous ethanol was used as the control in place of the DPPH-ethanol solution, and distilled water was used as the blank control in place of the polysaccharides solution. A V_C_ solution at the same concentration was used as the positive control, and the same procedure was repeated.

#### **2.8.2** **Hydroxyl (**OH**) radical scavenging activity**

The OH radical scavenging activity was evaluated using the method of Homayouni-Tabrizi et al. [33] with slight modifications. A 2.0 mL polysaccharides solution with varying concentrations was added to test tubes. To each test tube, 1.0 mL of 5.0 mmol/L FeSO4 solution and 1.0 mL of 5.0 mmol/L salicylic acid solution were added, followed by thorough mixing. Subsequently, 1.0 mL of 5.0 mmol/L H_2_O_2_ solution and 2.0 mL of distilled water were added, and the mixture was shaken well. The reaction was incubated in a water bath at 30 °C for 30 min. After the reaction, the absorbance was measured at 510 nm. Distilled water was used as a control instead of H_2_O_2_ solution, and as the blank control in place of the polysaccharides solution.

#### 2.8.3 Fe^3+^ reducing power

The determination of reducing power was referenced from the method of Fang Chen et al. [34] with minor modifications. A 2.0 mL sample solution with various gradient concentrations were added to test tubes. To each test tube, 2.0 mL of 1 % potassium ferricyanide solution and 2.0 mL of 0.2 mol/L phosphate buffer solution were added. The mixture was incubated in a water bath at 50 °C for 20 min, cooled to room temperature, and 2.0 mL of 10 % trichloroacetic acid solution was added. The reaction was allowed to proceed in the dark for 10 min, followed by centrifugation at 4000 rpm for 15 min. After centrifugation, 2.0 mL of the supernatant was mixed with 2.0 mL of distilled water and 0.2 mL of 1 % ferric chloride solution. The absorbance was measured at 710 nm. Distilled water was used as the blank control in place of the polysaccharides solution, and V_C_ with the same gradient concentrations was used as the positive control.

#### 2.8.4 ABTS^+^· radical scavenging activity

The determination method was based on the method of Irina Georgiana Munteanu, Constantin Apetrei [35]with slight modifications.

Equal volumes of 7.0 mmol/L ABTS solution and 2.5 mmol/L potassium persulfate solution were mixed and incubated in the dark for 12 h. The resulting solution was diluted with anhydrous ethanol to achieve an absorbance of 0.7 ± 0.02 at 734 nm. In each test tube, 0.5 mL of sample solution at varying concentrations was mixed with 4.5 mL of ABTS solution. The mixture was incubated at room temperature in the dark for 6 min. Absorbance was measured at 734 nm and recorded. Anhydrous ethanol was used as a control in place of the ABTS, and distilled water was used as the blank control in place of the sample solution.

### 2.9 α-Amylase inhibitory activity of *T. molitor* polysaccharides

The method for determining the α-amylase inhibitory activity of polysaccharides was based on Wang Xin [36] with minor modifications. Purified polysaccharides samples were prepared into solutions with concentrations of 0.2, 0.4, 0.6, 0.8, and 1.0 mg/mL.

A_1_: 0.4 mL of α-amylase solution (10 U/mL) was added to a test tube, followed by 0.2 mL of polysaccharides solution at each gradient concentration. The mixture was incubated in a 37 °C water bath for 10 min. After incubation, 0.3 mL of 1 % starch solution was added, and the mixture was further incubated at 37 °C for 15 min. The reaction was stopped by adding 0.2 mL of DNS reagent, and the test tube was boiled in a water bath for 10 min. After cooling, the volume was adjusted to 5 mL with distilled water, and the absorbance was measured at 540 nm. The procedure was repeated using acarbose as a positive control. The α-amylase inhibitory activity of the sample and acarbose was calculated according to Equation (3).

A_2_: The same procedure was repeated with distilled water replacing the α-amylase solution as the control.

A_0_: The same procedure was repeated with distilled water replacing the polysaccharides sample as the blank control.

(3) $$\alpha ‐\text{Amylase}\;\text{inhibition}\;\text{Rate}\left(\%\right)=1‐\frac{A_{1}‐A_{2}}{A_{0}}\times 100$$

### 2.10 Statistical analyses

The experimental results were expressed as the mean ± standard deviation, with experiments repeated three times. Analysis of variance (ANOVA) was performed, considering a p-value of less than 0.05 as statistically significant. Polynomial model fitting and optimization of conditions were conducted using Design-Expert 8.0. Data analysis and image processing were performed with Origin software (Origin 2018, OriginLab, Northampton, USA).

## 3 Results and analysis

### 3.1 Establishment of glucose standard curve

As shown in Fig. 2 (supplementary file), the linear regression equation for the standard curve was y = 13.685x+0.0712y = 13.685x + 0.0712y, with a correlation coefficient (R^2^) of 0.99698. This indicated a strong linear relationship between the glucose standard sample concentration and absorbance.

### 3.2 Screening results and property analysis of the DES system

#### 3.2.1 Screening results and analysis of the DES system

**Fig. 3:**
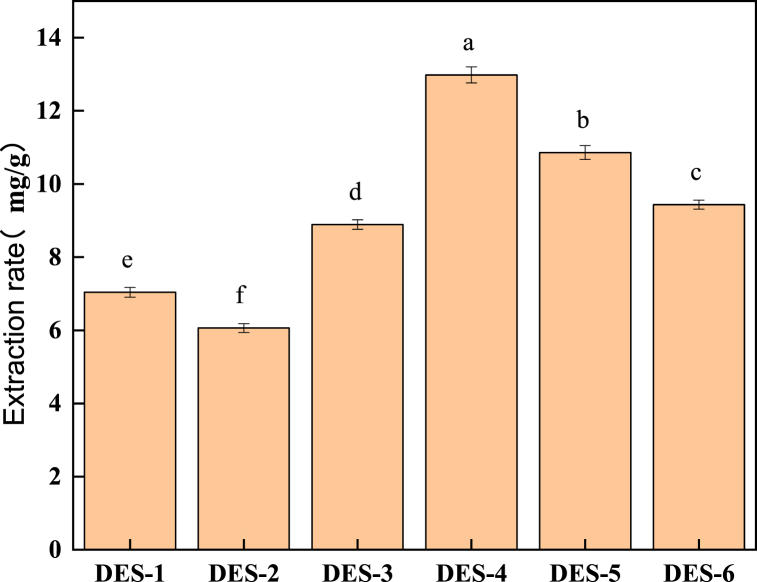
Yield of *T. molitor* polysaccharides in different DES systems.

Fig. 3 showed that a DES system composed of choline chloride and six different hydrogen bond donors was selected, with the yield of *T. molitor* polysaccharides as the response variable. The experimental results indicated that choline chloride-lactic acid produced the highest yield, 12.98 %, under identical conditions compared to other DES types. Therefore, choline chloride-lactic acid was chosen for the extraction of *T. molitor* polysaccharides in this study.

**Fig. 4:**
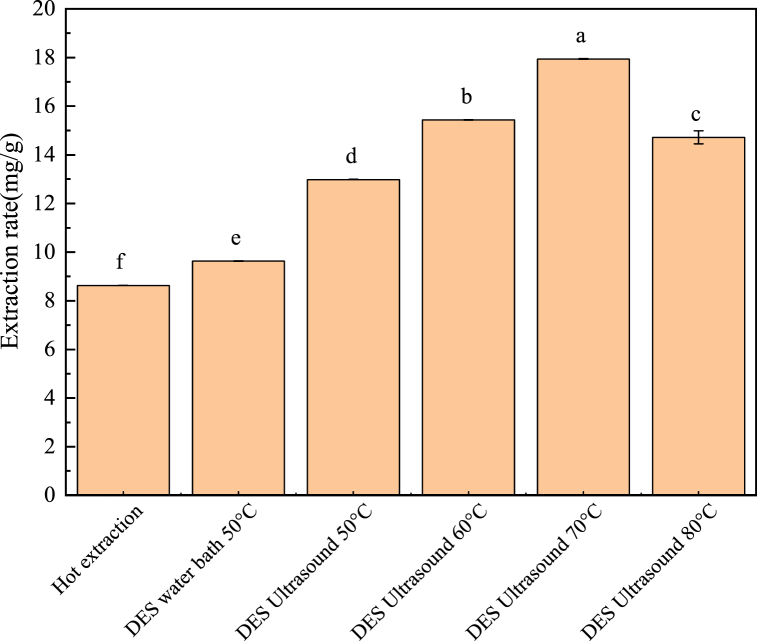
Yield of *T. molitor* polysaccharides under different extraction methods and process parameters.

Fig. 4 showed that, in the screening of extraction process parameters, the polysaccharides was yield of 9.69 % was obtained using water as the extractant under identical conditions. The highest yield, 17.98 % was achieved with ultrasound-assisted DES extraction at 70 °C. Consequently, 70 °C ultrasound-assisted DES extraction was selected as the fixed condition for the single-factor experiments.

#### 3.2.2 Results and analysis of DES physical property determination

Table 3 indicated that the physicochemical properties of DESs formed with different hydrogen bond donors and acceptors varied significantly, likely due to intramolecular hydrogen bonding interactions. This may have been due to the intramolecular hydrogen bonding interactions, and the formation of hydrogen bonds significantly affected quantum chemical parameters such as the chemical potential, hardness, softness, and electrophilic index of DES. These properties directly determined the performance of solvents in electron transfer processes and electrochemical applications [37]. These DESs were not bonded through covalent or ionic interactions [38], but were stabilized by intermolecular forces. Differences in pH arose from the inherent properties of the hydrogen bond donors; for instance, when the donors were acidic, the synthesized DESs tend to be acidic as well. The extensive hydrogen bonding networks among the components in the DESs led to poor fluidity and high viscosity. Additionally, electrostatic interactions and van der Waals forces contribute to the increased viscosity [39]. Most DESs exhibited densities ranging from 1.0 to 1.35 g/cm³ at 298.15 K [40], indicating that their densities were generally higher than that of water.

**Table 3:** Physicochemical properties of different DES.

typology	pH	Viscosity (mPa-S)	refractive index	Conductivity (ms/cm)	density
DES-1	8.90 ± 0.02	18.90 ± 0.21	1.451 ± 0.012	41.20 ± 0.47	1.1421 ± 0.0036
DES-2	2.90 ± 0.03	30.00 ± 0.11	1.443 ± 0.009	18.47 ± 0.33	1.1162 ± 0.0127
DES-3	5.16 ± 0.02	194.50 ± 0.06	1.449 ± 0.037	3.15 ± 0.14	1.0780 ± 0.0089
DES-4	2.53 ± 0.11	27.89 ± 0.15	1.440 ± 0.101	28.73 ± 0.08	1.1121 ± 0.1021
DES-5	0.98 ± 0.09	293.50 ± 0.18	1.451 ± 0.075	2.52 ± 0.16	1.3016 ± 0.0050
DES-6	1.23 ± 0.15	22.00 ± 0.09	1.436 ± 0.047	45.21 ± 0.22	1.1640 ± 0.0053

### 3.3 Results and analysis of single -factor experiment for extracting *T. molitor* polysaccharides using the DESs

#### 3.3.1 Effect of DES water content on polysaccharides yield

**Fig. 5:**
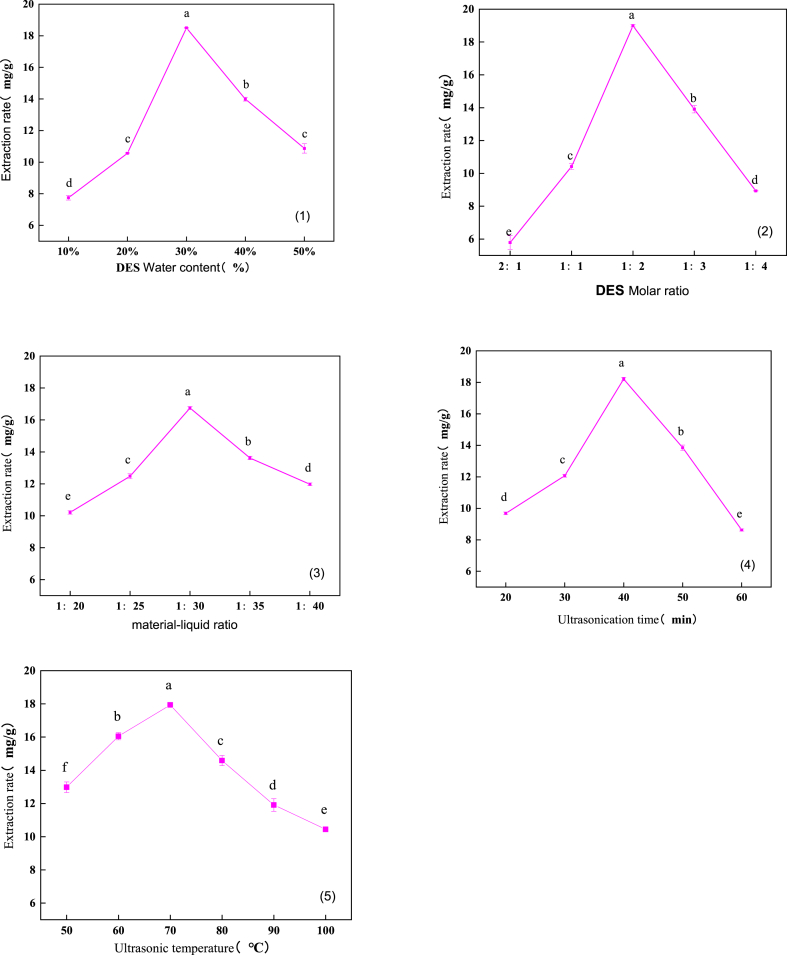
Effect of DES water content、molar ratio、material-liquid ratio、ultrasonic time、ultrasonic temperature.

Water content had a significant impact on the extraction efficiency of DES; it not only increased the polarity, solubility, and mass transfer capacity of DES but also reduced its viscosity and surface tension [41]. As shown in Fig. 5(1), the extraction efficiency varied with water content, reaching a maximum of 18.50 % at 30 % water content. However, excessive water could severely damage the internal structure of DES [42] and weaken the interactions between its components, thereby affecting the extraction performance. Therefore, a water content of 30 % was determined to be optimal. ANOVA indicated a P-value of less than 0.05 for this factor, confirming its significant effect.

#### 3.3.2 Effect of DES molar ratio on the yield of polysaccharides

As shown in Fig. 5(2), the polysaccharides yield exhibited a significant trend of increasing and then decreasing as the molar ratio of choline chloride to lactic acid was varied. The highest yield of 18.19 % was achieved at a DES molar ratio of 1:2. This result was attributed to the high viscosity and poor solubility of the solvent at a molar ratio of 2:1, which impeded polysaccharides dissolution. As the molar ratio increased, the viscosity and surface tension of the DES decreased, and solubility improved. However, further increases in the molar ratio led to a decline in polysaccharides yield, likely due to the weakening of interactions between the components when the hydrogen bond donor content was excessively high [43]. The results indicate that a DES molar ratio of 1:2 was optimal. ANOVA yielded a p-value of less than 0.05 for this factor, confirming its significance.

#### 3.3.3 Effect of material-liquid ratio on the yield of polysaccharides

As shown in Fig. 5(3), the yield of polysaccharides gradually increased with time. At 40 min, the maximum yield of 17.91 % was achieved. However, beyond 40 min, the yield began to decline rapidly [44]. This decline may be attributed to the mechanical damage caused by ultrasonic treatment, as prolonged ultrasonic exposure can lead to the destruction of polysaccharides molecules. Therefore, 40 min was identified as the optimal extraction duration. This factor was significant, with P < 0.05, as determined by the analysis of variance.

#### 3.3.4 Effects of ultrasound time on polysaccharides yield

As shown in Fig. 5(4), the yield of polysaccharides gradually increased with time, reaching a maximum of 17.91 % at 40 min. Beyond this point, the yield declined rapidly [45]. This decline may be attributed to mechanical damage to polysaccharides caused by prolonged ultrasonic treatment, which can degrade polysaccharides molecules. Therefore, 40 min was identified as the optimal extraction duration. Analysis of variance confirmed the significance of this factor, with a p-value less than 0.05.

#### 3.3.5 Effects of ultrasound temperatures on polysaccharides yield

Research has shown that high temperatures can reduce the viscosity of DES, increase solubility, and accelerate the movement of target molecules [46]. As shown in Fig. 5(5), within the temperature range of 50–70 °C, the yield of polysaccharides increased rapidly with rising temperature, aligning with the aforementioned findings. However, when the temperature exceeded 80 °C, the yield began to decline, likely due to the oxidation of polysaccharides at high temperatures, resulting in reduced yield. Therefore, the optimal extraction temperature for *T. molitor* polysaccharides was determined to be 70 °C. Analysis of variance revealed that this factor was not significant, with a p-value greater than 0.05.

### 3.4 Response surface design and results

#### 3.4.1 Establishment and analysis of the regression model for the yield of *Tenebrio molitor* polysaccharides

Based on the results of the single-factor experiment, molar ratio, ultrasonic time, and water content were investigated as variables, with polysaccharides yield as the response variable. The regression equation derived from the software analysis was: y = 18.65 + 1.37 × A+1.19 × B-0.97 × C-0.84 × A × B- 0.42 × A × C+0. 085 × B × C-1.74 × A^2^-4.07 × B^2^-2.15 × C^2^, Variance analysis was conducted on the 17 experimental groups, as shown in Table 4**.**

**Table 4:** Response surface test design and results.

No.	A	B	C	yield(%)
1	0	0	0	18.89 ± 0.34
2	1	−1	0	15.23 ± 0.21
3	0	0	0	18.45 ± 0.31
4	−1	0	−1	13.59 ± 0.17
5	0	1	1	12.12 ± 0.22
6	0	−1	−1	12.33 ± 0.29
7	1	1	0	8.18 ± 0.18
8	1	0	1	14.50 ± 0.21
9	0	0	0	18.83 ± 0.19
10	0	0	0	18.84 ± 0.21
11	0	1	−1	10.16 ± 0.17
12	−1	0	1	16.51 ± 0.29
13	1	0	−1	13.25 ± 0.22
14	0	0	0	16.77 ± 0.31
15	0	−1	1	13.96 ± 0.19
16	−1	1	0	14.15 ± 0.17
17	1	−1	0	12.62 ± 0.27

From Table 5, it was observed that the P-value of the regression model was 0.0011, which was less than 0.01, indicating that the regression model for extracting polysaccharides from *T. molitor* using ultrasonic-assisted DES was highly significant. The P-value of the lack of fit term was 2.11, which was greater than 0.05, suggesting that the difference between the calculated and experimental results of the regression model was not significant. Based on the P-values and F-values, the impact on polysaccharides yield from *T. molitor* was ranked as follows: molar ratio > ultrasonic time > water content. Among these factors, the effects of terms A, A^2^, B^2^, and C^2^ were extremely significant, while the effects of terms B and AB were significant. This indicated that the model was suitable for optimizing the polysaccharides extraction process.

**Table 5:** ANOVA of the regression equation.

Source	Sum Of Squares	df	Mean Of Square	F Value	PValue	Significant
Model	149.06	9	16.56	13.76	0.0011	∗∗
X_1_	7.54	1	7.54	6.26	0.0181	∗
X_2_	14.95	1	14.95	12.42	0.0097	∗∗
X_3_	11.34	1	11.34	9.42	0.0409	∗
X_1_X_2_	2.84	1	2.84	2.36	0.1683	
X_1_X_3_	0.70	1	0.70	0.58	0.4719	
X_2_X_3_	0.029	1	0.029	0.024	0.8818	
X_1_^2^	12.81	1	12.81	10.64	0.0138	∗
X_2_^2^	69.61	1	69.61	57.81	0.0001	∗∗
X_3_^2^	19.44	1	19.44	16.14	0.0051	∗∗
Residual	8.43	7	1.20			
Lack Of Fit	5.17	3	1.72	2.11	0.2414	
Pure Error	3.26	4	0.82			
Cor Total	157.49	16				

∗ Significant (P < 0.05). ∗∗ Highly significant (P < 0.01).

#### 3.4.2 Response surface analyses of the yield of *T. molitor* polysaccharides

**Fig. 6:**
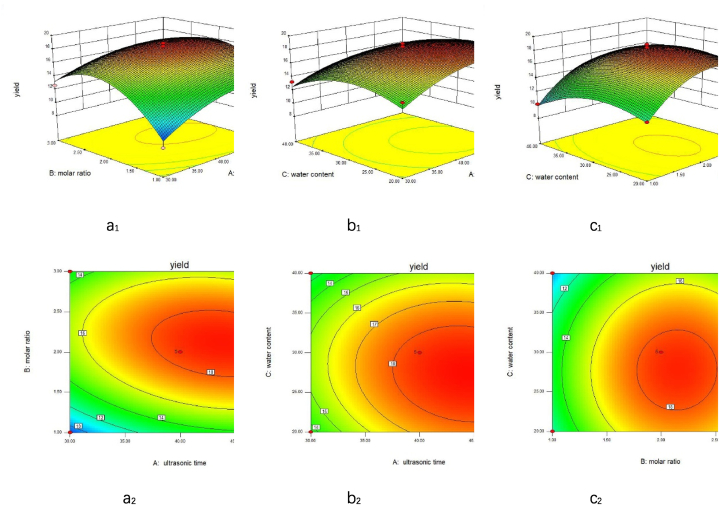
Response Surface and Contour Plots: Ultrasonic Time and Molar Ratio (a), Ultrasonic Time and Water Content (b), Molar Ratio and Water Content (c) Subscripts 1 and 2 are the response surface contour maps, respectively.

Fig. 6(a_1_、a_2_), 6(b_1_、b_2_), and 6(c_1_、c_2_) illustrated that all three response surface plots were downward-opening surfaces with distinct peaks. The steeper the response surface curve, the more pronounced the factor's effect on the polysaccharides yield of *T. molitor.* From the curvature of the response surface in Fig. 6(a_1_), it was evident that, with constant sonication time, the effect of varying the molar ratio on polysaccharides yield initially increased and then decreased, showing a clear trend. When the molar ratio was constant, the trend of sonication time on polysaccharides yield was more pronounced, indicating that the molar ratio had a greater impact on yield compared to sonication time. Similarly, Fig. 6(b_1_) demonstrated that sonication time had a more substantial effect on polysaccharides yield than water content, while Fig. 6(c_1_) showed that the molar ratio had a greater influence on yield than water content. Based on the regression model analysis, the optimal conditions for ultrasound-assisted DES extraction of polysaccharides from *T. molitor* were as follows: a DES molar ratio of 1:2.1, a water content of 27.36 %, an extraction temperature of 70 °C, and an extraction time of 43.99 min, resulting in polysaccharides yield of 18.82 %. After adjusting these conditions to a DES molar ratio of 1:2.1, a water content of 27 %, an extraction time of 44 min, and an extraction temperature of 70 °C, three parallel experiments were performed. The average polysaccharides yield was 18.62 %. The yield obtained under these adjusted conditions closely matched the software prediction, confirming the validity of the regression model's predicted conditions. Similar studies were done by Zubera Naseem et al. [47].

### 3.5 Infrared spectral analysis

**Fig. 7:**
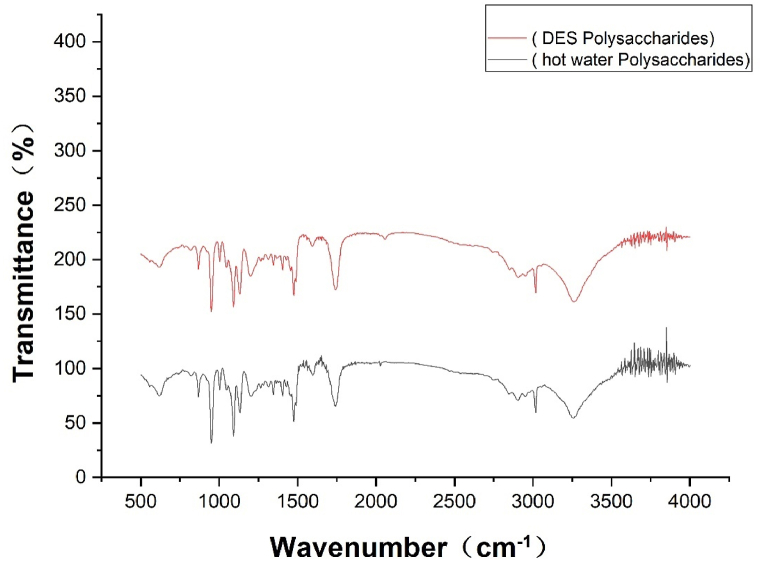
Infrared spectroscopy of mealworm polysaccharides.

Infrared spectroscopy is an extremely important method in the structural analysis of polysaccharides to characterize the chemical structure of polysaccharides at specific wave numbers [48]. As shown in Fig. 7, the signals at 3300, 2920, 1750, 1490, and 1100 cm^−1^ were typical of polysaccharides [49]. The strong absorption peak located near 3300 cm^−1^ was derived from the stretching vibration of the O-H group [50]. The signal at 2922 cm^−1^ was caused by the C-H stretching vibration. The strong absorption peak near 1750 cm^−1^ indicated the stretching vibration of C=O. The absorption near 1490 cm^−1^ was due to the deformation vibration of C-H [51]. Absorption of C-O-C groups usually occurs near 1000-1100 cm-1 suggesting that these polysaccharides were composed of a pyranose ring [52]. The 875 cm^−1^ the weaker signal near represents the vibration of β-glycosidic bonds, while the absence of absorption peaks near 750 cm^−1^ indicated the absence of α-glycosidic bonds in these polysaccharides [53]. The Infrared spectral results showed that the hot water extracted polysaccharides and DES extracted polysaccharides had similar structures and were composed of pyranose linked by β-glycosidic bonds. Similar studies were done by Jixian Zhang et al. [54].

### 3.6 Results and analysis of inhibitory properties of *T. molitor* polysaccharides

#### 3.6.1 The determined of MIC

As shown in Table 6, using the filter paper diffusion method, *T. molitor* polysaccharides exhibited inhibitory effects on four bacterial species, but not on fungi. The minimum inhibitory concentrations (MIC) for *Staphylococcus aureus*, *Escherichia coli*, *Salmonella*, and *Bacillus subtilis* were 1, 2, 0.5, and 2 mg/mL, respectively.

**Table 6:** Diameter of inhibition zone of *T. molitor* polysaccharides (cm).

	concentration (mg/ml)						
	10	8	6	4	2	1	0.5
S**taphylococcus aureus**	1.58	1.46	1.34	1.26	1.10	–	–
E**scherichia coli**	1.34	1.22	1.18	1.08	–	–	–
S**almonella**	2.06	1.84	1.62	1.34	1.10	1.08	–
B**acillus subtilis**	1.59	1.46	1.26	1.18	–	–	–
Y**east**	–	–	–	–	–	–	–
**Aspergillus niger**	–	–	–	–	–	–	–

(∗ The diameter of the filter paper discs is 9 mm, and the measurements include the diameter of the filter paper discs).

#### 3.6.2 Antibacterial properties

By determining the MIC, it was concluded that *T. molitor* polysaccharides exhibit stronger inhibition against Gram-negative bacteria compared to Gram-positive bacteria, with minimal inhibition observed on fungi. As shown in Table 6, *T. molitor* polysaccharides demonstrated the most significant inhibition against *Salmonella*. Experimental data indicated that the inhibitory effects of the polysaccharides on common bacteria, ranked from strongest to weakest, were *Salmonella*, *Staphylococcus aureus*, *Escherichia coli*, and *Bacillus subtilis*.

### 3.7 Antioxidant activity of *Tenebrio molitor* polysaccharides

#### **3.7.1** DPPH radical scavenging activity of *T. molitor* polysaccharides

**Fig. 8:**
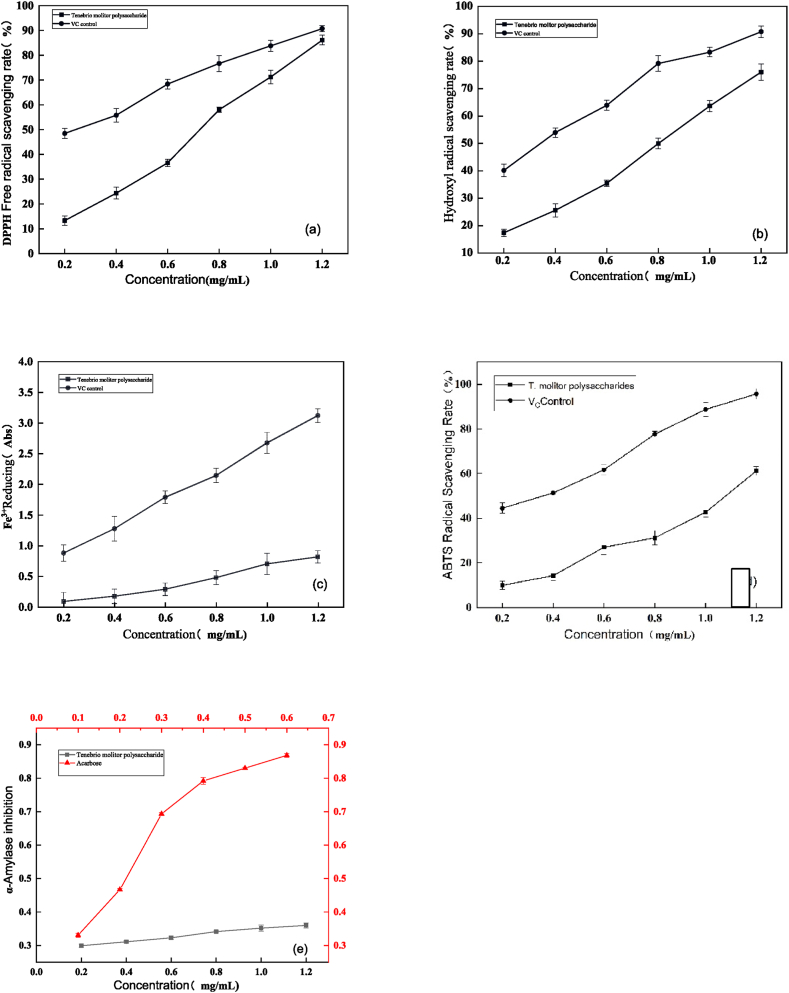
DPPH·(a)、OH·(b) and ABTS(d) scavenging ability of hydrolysates Fe3+ Reduction Capacity(c), a-enzyme inhibitor(e).

As shown in Fig. 8(a), the scavenging effect of polysaccharides from *T. molitor* on DPPH· increased progressively with higher concentrations. When the mass concentration of polysaccharides from 0.2 to 1.2 mg/mL, the scavenging rate varied from 13.42 % to 86.11 %. At the V_C_ concentration of 1.2 mg/mL, the DPPH• scavenging rate achieved 90.73 %. The IC_50_ values of polysaccharides and V_C_ in this experiment were calculated to be 0.6225 mg/mL and 0.2640 mg/mL, respectively.

#### **3.7.2** Hydroxyl radical scavenging activity of *T. molitor* polysaccharides

As shown in Fig. 8(b), the hydroxyl radical scavenging ability of the polysaccharides of *T. molitor* was positively correlated with the concentration, though it was lower than that of V_C_. The scavenging rate of hydroxyl radicals ranged from 17.42 % to 76.04 % for polysaccharides concentrations ranging from 0.2 to 1.2 mg/mL. The IC_50_ values for *T. molitor* polysaccharides and the V_C_ control group were calculated to be 0.6930 mg/mL and 0.3196 mg/mL, respectively.

#### **3.7.3** Reducing power of *T. molitor* polysaccharides toward Fe³⁺

There was a relationship between the reducing power of the sample and its antioxidant activity, where the sample's ability to generate electrons or hydrogen ions through the reaction of reducing force helps to neutralize free radicals. A greater reducing power indicates stronger antioxidant capacity. As can be seen in Fig. 8(c) –*T molitor* polysaccharides exhibited a certain degree of reducing power. With increasing concentration, the absorbance ranged from 0.087085 to 0.815205, indicating a continuous enhancement in reducing power. However, compared to the reducing power of V_C_ in the positive control group, the reducing power of mealworm polysaccharides was weaker than that of V_C_.

#### **3.7.4** ABTS⁺ radical scavenging activity of *T. molitor* polysaccharides

As shown in Fig. 8(d), when the mass concentration ranged from 0.2 to 1.2 mg/mL, the scavenging ability of the polysaccharides on ABTS^+^• free radicals increased with concentration, with the scavenging rate ranging from 9.56 % to 61.02 %. Although the polysaccharides exhibited relatively low scavenging ability, the V_C_ group achieved a scavenging rate of 44.3 %, demonstrating the effectiveness of the control group. The IC_50_ values for the polysaccharides and V_C_ in the positive control group were 1.0955 and 0.3264 mg/mL, respectively.

### 3.8 α-amylase inhibitory activity

As shown in Fig. 8(e), at a polysaccharides concentration of 0.2 mg/mL, the α-amylase inhibition rate reached 29.93 %. When the concentration was increased to 1.0 mg/mL, the inhibition rate rose to 35.12 %. In comparison, acarbose, used as a positive control, exhibited a stronger inhibitory effect, with an inhibition rate of 86.25 %.

## 4 Conclusions

This study focused on the polysaccharides extracted from *T. molitor*, using ultrasound-assisted DES. Various DESs were screened, and the polysaccharides extraction process was optimized through response surface methodology. The antibacterial, antioxidant, α-amylase inhibitory activities, and structural characteristics of the polysaccharides were also investigated. The conclusions were as follows: Significant differences were observed in pH, viscosity, and conductivity among the six DESs, while differences in refractive index and density were not significant. Choline chloride-lactic acid was identified as the optimal extraction agent. The optimal extraction parameters for *T. molitor* polysaccharides, determined using response surface methodology, were: a DES molar ratio of 1:2.1, a water content of 27 %, an extraction temperature of 70 °C, and an extraction time of 44 min, resulting in polysaccharides yield of 18.62 %. The polysaccharides from *T. molitor* demonstrated inhibitory effects against four common pathogens, as well as *Aspergillus niger* and *yeast,* with the most significant inhibition observed against *Salmonella*. The MIC of the polysaccharides against *Bacillus subtilis*, *Escherichia coli*, *Staphylococcus aureus*, and *Salmonella* were 2, 2, 1, and 0.5 mg/mL, respectively. Within a certain concentration range, the polysaccharides exhibited strong scavenging activity against DPPH• and ABTS+• free radicals, with IC_50_ values of 0.6225, 1.0955, and 0.6930 mg/mL, respectively. Additionally, the polysaccharides demonstrated a certain degree of reducing ability toward Fe^3+^. As the sample concentration increased, its antioxidant capacity also improved. The polysaccharides also demonstrated inhibitory activity against α-amylase, with an IC_50_ value of 8.702 × 10^1^⁷ mg/mL, and the inhibitory activity increased with concentration. Infrared spectroscopy analysis revealed the presence of functional groups such as C-O, O-H, and C=O confirming the substance as polysaccharides molecule. Natural polysaccharides exhibit significant biological activity, making them valuable for human health and industrial applications. This study provides a theoretical reference for the development and utilization of low-consumption, large-scale biological resources such as *T. molitor*. Future research should focus on further purifying the polysaccharide extract to accurately determine its biological activity and structural characteristics.

## CRediT authorship contribution statement

**Shengru Yang:** Writing – review & editing, Writing – original draft. **Xu Li:** Methodology. **Qiaoli Li:** Data curation.

## Data availability statement

The datasets used and/or analyzed during the current study are available from the corresponding author on reasonable request.

## Ethics declaration

Review and/or approval by an ethics committee as well as informed consent was not required for this study because this article did not involve any direct experimentation/studies on living beings.

## Declaration of competing interest

The authors declare the following financial interests/personal relationships which may be considered as potential competing interests:Reports a relationship with that includes:. Has patent pending to. If there are other authors, they declare that they have no known competing financial interests or personal relationships that could have appeared to influence the work reported in this paper.
