# Supplementary material for: Deep eutectic solvent extraction and biological activity of polysaccharides from Tenebrio molitor
Source: Heliyon. 2025 Jan 11;11(2):e41790. doi: 10.1016/j.heliyon.2025.e41790 (PMC11786816; doi:10.1016/j.heliyon.2025.e41790)
Supplement: Multimedia component 1 [file mmc1.docx]

Figure. 2 Standard curve of glucose
